# Molecular Markers of Agronomic Traits and Superior Germplasm of Spring Wheat in Northern Kazakhstan and Siberia

**DOI:** 10.3390/ijms27219547

**Published:** 2026-10-26

**Authors:** Anastasiya Chernook, Mikhail Divashuk, Aleksey Ermolaev, Vladimir Shamanin, Timur Savin, Tatyana Shelaeva, Vladimir Chudinov, Elena Fedorenko, Sergey Shepelev, Daniyar Tajibayev, Alexey Morgounov

**Affiliations:** 1 All-Russia Research Institute of Agricultural Biotechnology, Moscow 127550, Russia; divashuk@gmail.com (M.D.); ermol-2012@yandex.ru (A.E.); alexey.morgounov@gmail.com (A.M.); 2 Faculty of Agrotechnology, Omsk State Agrarian University, Omsk 644008, Russia; vp.shamanin@omgau.org (V.S.); ss.shepelev@omgau.org (S.S.); 3 Research Department, Stev Agro, Astana 010000, Kazakhstan; savintimur_83@mail.ru; 4 A.I. Barayev Research and Production Centre for Grain Farming, Shortandy 021601, Kazakhstan; tatyana.shelaewa@yandex.kz; 5 Karabalyk Agricultural Experimental Station, Kostanay 110000, Kazakhstan; ch.den@mail.ru; 6 North Kazakhstan Agricultural Experimental Station, Shagalaly 150311, Kazakhstan; efedorenko2015@mail.ru; 7 Independent Researcher, Astana 010000, Kazakhstan; daniyar.taj@gmail.com

**Keywords:** spring wheat, genome-wide association study (GWAS), single nucleotide polymorphism (SNP), grain yield, plant height, heading date, marker-assisted selection

## Abstract

Spring bread wheat (*Triticum aestivum* L.) is the principal crop of the vast rainfed region spanning Northern Kazakhstan, the Urals, and Western Siberia, where productivity is limited by moisture stress, diseases, and the slow adoption of genomic tools in traditional breeding. We performed a genome-wide association study (GWAS) of 142 spring wheat genotypes from the Kazakhstan–Siberia Network for Spring Wheat Improvement (KASIB), evaluated at four sites in Russia and Kazakhstan during 2020–2022. The analysis identified 23 significant marker–trait associations (SNPs), of which 21 were stable across at least two environments, and 11 showed pleiotropic effects on two or more traits. Grain yield was associated with two SNPs (1A_12366171_G_A and 1B_15284504_C_T), each also affecting heading and maturity. Heading and maturity were controlled by a cluster of SNPs on chromosomes 1B, 2B, 5A, and 7B, with effects of up to 5.1 days. Plant height was associated with SNPs on 1A, 5A, and 6B, including one marker (5A_585412831_A_G) that was pleiotropic with 1000-kernel weight. Spike productivity was associated with loci on 1A, 3D, 4A, 4D, 5B, and 7B (grain weight and grains per spike, spikelets per spike, and spike harvest index). Grain size and 1000-kernel weight were linked to SNPs on 1B, 5A, and 6B, and protein and gluten content to two loci on 3A and 4B, which raised protein by up to 0.62% and gluten by up to 1.39%.

## 1. Introduction

Spring wheat production regions across North Kazakhstan, the Urals and Western Siberia in Russia cover more than 17 million hectares. The crop is normally planted in May and harvested between August and September. It forms part of a broader production system that also includes other cereals (spring durum wheat, barley, oats), oilseed crops (sunflower, rapeseed, flax) and legumes (dry peas, lentils, chickpeas). Because the crop is grown entirely under rainfed conditions, moisture stress represents the principal abiotic constraint on grain yield, while diseases such as leaf and stem rust, Septoria species and tan spot, along with a range of insect pests, recur regularly and further limit production. Average regional grain yields fluctuate between 1.2 and 1.8 t/ha depending on the season [1], a shortfall attributable in part to reliance on low-input production practices and conventional tall-statured cultivars.

Wheat breeding in the region is carried out mainly through public programs based at agricultural research stations, institutes and centers distributed across the principal agro-ecological zones [2]. These programs rely on a breeding system built around crosses followed by selection through a modified pedigree method, with yield testing reserved for the more advanced breeding stages. Developing a new variety typically requires 10–12 years, with an additional 2–3 years needed for official registration trials and a further 3–5 years for kernel multiplication [2]. Few programs in the region have adopted modern genomic or phenomic tools, and multilocational trials at advanced breeding stages remain scarce. Consequently, many released cultivars remain disease-susceptible, tall and prone to lodging under favorable conditions, and poorly responsive to inputs. At the same time, European cultivars have gained ground among farmers thanks to their shorter stature, which makes them more compatible with modern technology. These circumstances point to a clear need to modernize regional breeding programs, including through the adoption of genomic and phenomic approaches.

DNA molecular markers have become a valuable tool for selecting genotypes carrying desirable traits [3]. This rapidly advancing technology enables highly efficient selection for a wide array of wheat characteristics, including resistance to biotic and abiotic stresses, adaptability, agronomic performance and grain quality. Recent reviews by Singh et al. [4] and Gupta [5] provide a comprehensive summary of the various wheat molecular markers and their applications in breeding. These include drought tolerance and yield potential improvement using diverse genomic tools. Within North Kazakhstan and Western Siberia specifically, research into the genetics and molecular marker identification of spring wheat has centered on grain elemental composition [6], leaf rust resistance [7] and agronomic traits [8,9]; however, none of these findings have yet been translated into applied breeding practice. The scientific questions which remain unresolved include identification of genetic markers associated with adaptation and productivity traits across the Eurasian spring wheat belt. These include days to heading and plant height, spike productivity traits and grain yield. Genome-wide association mapping (GWAS) proved to be a reliable tool for this purpose [8,9].

Founded in 2000, the Kazakhstan–Siberia Network for Spring Wheat Improvement (KASIB) brings together more than 15 breeding and research programs with the shared goal of exchanging germplasm and conducting joint multilocational trials to evaluate advanced breeding lines and new cultivars. Each member institution contributes 2–3 entries to a cooperative trial spanning two years, and the resulting data are compiled and shared across the network. By 2018, the network had exchanged and evaluated more than 300 genotypes through KASIB yield trials, from which a core set of 142 genotypes was subsequently selected. This core set was genotyped using common KASP markers and GBS and phenotyped over three years (2020–2022) at four institutions: Omsk State Agrarian University (Omsk SAU), Kazakh Scientific-Production Center of Grain Farming (Kaz. SPC Grain), Karabalyk Agricultural Experimental Station (AES) and North Kazakhstan AES. A previous analysis showed the effects of spring- and winter-type alleles of the *Vrn-A1* gene on heading date and grain yield [10]. Translocation of 1BL.1RS was present in 21.8% of the material, which resulted in a 6.2% yield advantage compared to 1B.1B germplasm and a reduction in stem rust severity from 27.6 to 6.6%. Several other markers significantly influenced 1000-kernel weight (1000KW) and grain yield. The aim of the present study was to conduct GWAS and identify genetic regions related to adaptation and productivity traits, with a view towards their practical application in breeding. Selection of superior high-yielding germplasm for utilization in breeding was also an important study objective.

## 2. Results

### 2.1. Grain Yield and Agronomic Traits Across the Study Sites and Years

**Figure 1:**
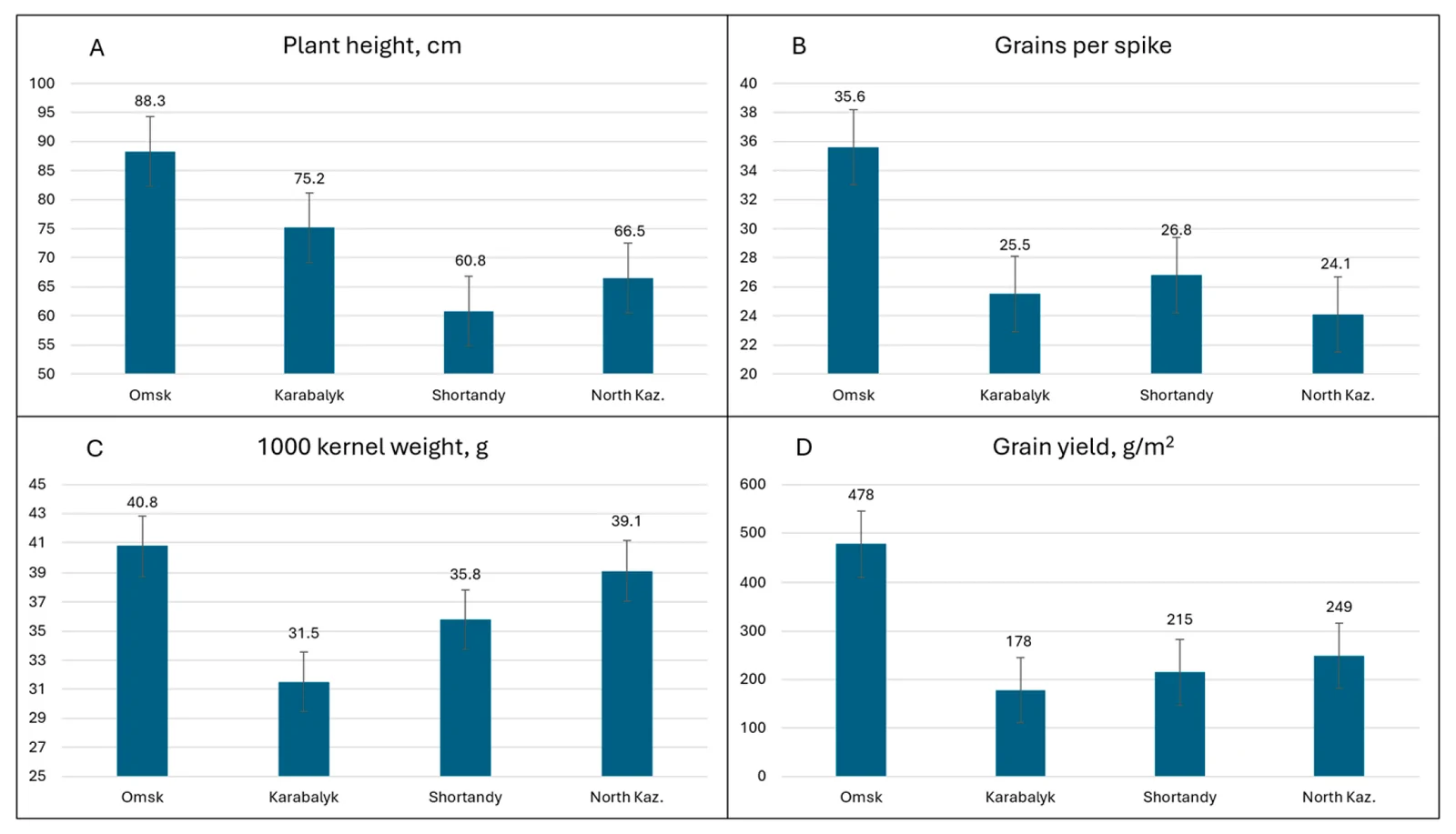
Average plant height (**A**), number of grains per spike (**B**), 1000-kernel weight (**C**) and grain yield (**D**) at four sites in 2020–22.

Mean grain yield and other agronomic traits across sites and common study years (2021–22) are presented in Figure 1 and Table S1. ANOVA revealed highly significant effects of site, year, and genotype, as well as genotype × site and site × year interactions, for all traits (plant height, spike length, grains per spike, grain weight per spike, 1000-kernel weight, and grain yield) (Table S2). The Omsk environment was the most favorable for spring wheat among the four sites, outperforming the three locations in Kazakhstan. Mean grain yield at Omsk reached 478 g/m^2^, compared with 178–249 g/m^2^ at the other sites, and plant height averaged 88.3 cm over the three years—21.8–27.5 cm taller than elsewhere. Among the yield components, grains per spike averaged 35.6 at Omsk versus 24.1–26.8 at the Kazakhstan sites. For 1000-kernel weight, however, Omsk only marginally exceeded the North Kazakhstan station (40.8 vs. 39.1 g), and the lowest 1000-kernel weight (TKW) was recorded at Karabalyk (31.5 g). Substantial variation for agronomic traits was observed between years (Table S1). At Kazakhstan sites, weather conditions in 2020 favored spring wheat, resulting in higher grain yield compared to 2020 and 2021. The period from seedling emergence to heading varied from 38 to 44 days depending on the site and year, while the period from heading to maturity varied from 30 to 40 days. Essentially, the growing season of spring wheat at experimental sites was very short—within 70–85 days. The coefficient of correlation between the number of days to heading and grain productivity across all sites and years was positive and highly significant (r = 0.37, *p* < 0.001), suggesting higher grain yield of later-developing genotypes.

### 2.2. GWAS Results

**Figure 2:**
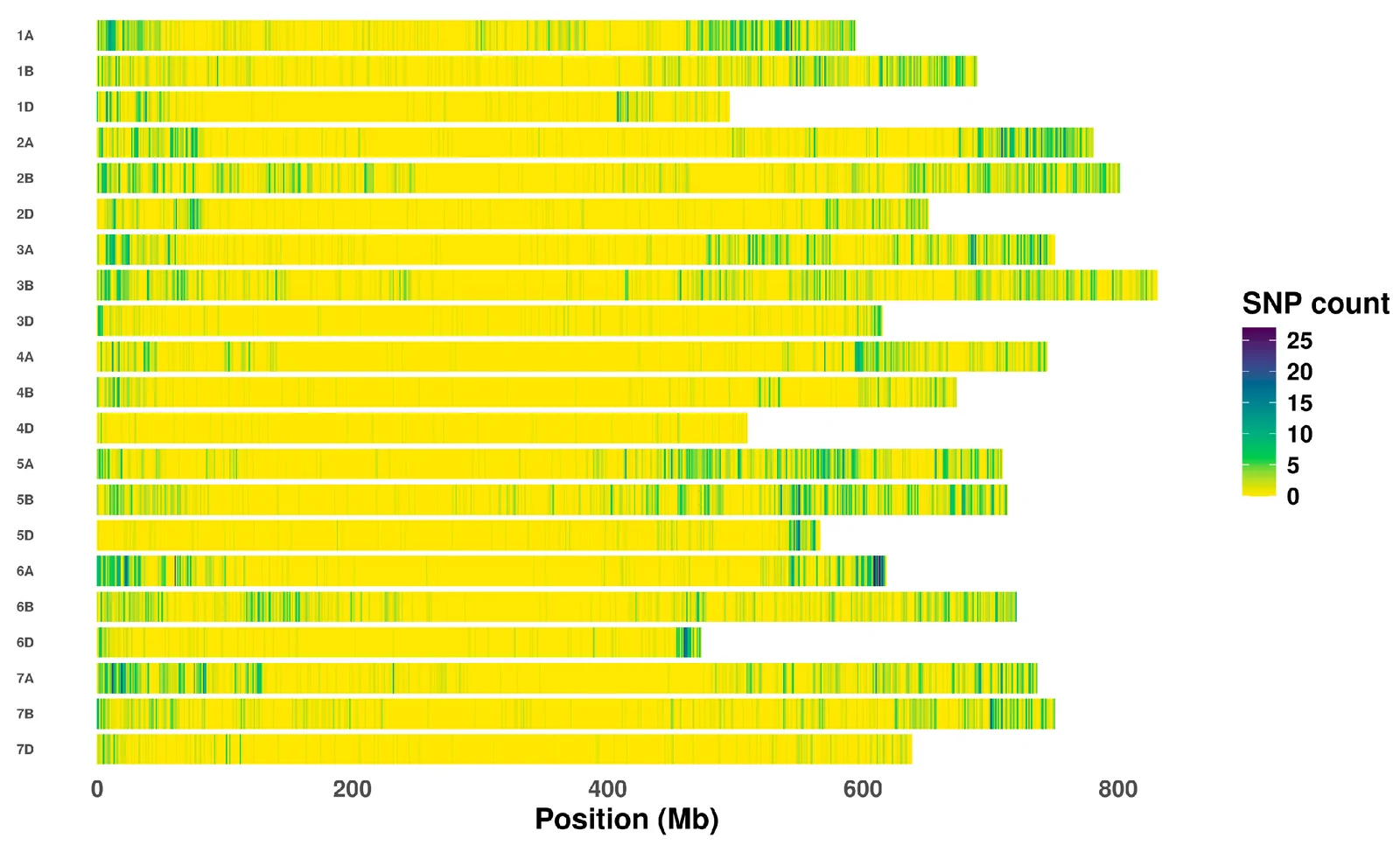
Distribution of genotyped variants after QC across bread wheat chromosomes.

The initial dataset consisted of 21,693 biallelic sites. Variants with a missing rate >5% were removed, leaving 18,240 variants (3453 discarded). Rare variants with a minor allele frequency (MAF) <8% were then removed, leaving 12,626 variants (5614 discarded). Finally, 124 variants with excessively high observed heterozygosity (>10%) were removed, leaving 12,502 variants for downstream imputation and annotation. The distribution of high-quality variants across chromosomes (Figure 2) is skewed toward the distal, gene-rich regions [11]. Due to the extensive volume of GWAS results, including Manhattan and QQ plots, all files were uploaded to Zenodo (https://zenodo.org/records/23162912) (accessed on 8 September 2026) [12].

Stability of the association across several ‘location × year’ combinations is a key criterion of a locus’s practical value for marker-assisted selection. Of the 23 SNPs, 21 showed stability (≥2 environments), and 11 showed a pleiotropic effect (≥2 traits) (Table 1). Among these 21, there are 7 loci that showed an effect in 4–6 sites simultaneously: 7B_9702496_G_C, the most stable locus in the entire dataset, detected in 6 instances (OMSK_2020, OMSK_2021, OMSK_2022, OMSK_ALLYEARS, ALLLOC_2021, ALLLOC_ALLYEARS) for the trait “spikelets per main spike” and in 1 instance for “days to heading.” The effect is positive (+0.36…+0.52 spikelets). Locus 1A_12366171_G_A showed stability in 5 instances (ALLLOC_2020, ALLLOC_ALLYEARS, KARABALYK_2020, KARABALYK_2022, KARABALYK_ALLYEARS) for the trait “days to maturity”; the allele shortens the growing season. Locus 5A_585020739_G_T is stable in 5 instances for the trait “days to heading” and in 1 instance for “plant height”; the allele increases the period to heading by 1.3–1.8 days and plant height by 3.10 cm. Locus 1A_539763488_C_T showed stability in 4 instances and affected 3 traits simultaneously (grain weight, spike weight, and grain number). All effects are positive. Locus 1A_13767380_A_G is stable in 4 instances (ALLLOC_2020, ALLLOC_ALLYEARS, OMSK_2022, OMSK_ALLYEARS) for the trait “plant height.” The allele reduces plant height by 2.56–4.60 cm. Locus 4D_509666790_G_A showed stability in 4 instances for the trait “spike harvest index” and in 1 instance for “grain width,” with a positive effect. Locus 6B_682548960_T_C is stable in 4 instances for the trait “1000 KW,” although the allele reduces grain weight.

### 2.3. SNP Effect Reproducibility

Replication of SNPs with significant effects (Table 1) was conducted using regular KASIB trial data from 2005 to 2018 across 10–12 locations in Kazakhstan and Russia. Since the KASIB core set was selected from previous trials, a large dataset on agronomic performance (grain yield, plant height, days to heading and 1000-kernel weight) was available from many sites and years where some entries of the core set were evaluated. SNP 1A_12366171 initially affected the number of days to maturity at five environments (Karabalyk and all sites in different years) (Table 1). During replication with a larger KASIB dataset, this SNP was significantly associated with days to heading at Karabalyk AES in 2010, grain yield at the same location in 2017 and grain yield at Omsk SAU (2014, 2016) and Chelyabinsk ARI (2017) (Table 2). SNP 1B_15284504 was identified as significantly associated with days to heading at Karabalyk AES in 2021 and 2020–22 and confirmed its association at the same site in 2010. In addition, this marker had significant association with grain yield at Omsk SAU in 2014. SNPs 5A_585412831 and 5A_586604587 are probably linked to the same gene, and in the GWAS panel the former was associated with plant height and 1000-kernel weight, while the latter—number of days from heading to maturity. In replication trials, their effect was expressed in number of days to heading in Kurgan, Aktobe and Altay in 2011. So replication of four markers using the same material but different environments confirms their effects on the same traits and also shows additional associations with other important agronomic traits. These markers are likely to be effectively used in breeding programs.

### 2.4. Performance of Superior Germplasm

The average values of grain yield, days to heading and 1000-kernel weight across all 11 trials for the highest-yielding entries in three maturity groups are presented in Table 3. Information on the entire studied collection is presented in Table S3. Presence of several markers in respective genotypes is also given: 1A_12366171 and 1B_15284504 (days to heading and grain yield), 1A_539763488 (spike productivity), 5A_585412831 (days to heading, plant height and 1000 KW), 6B_682548960 (1000KW), and 7B_9702496 (days to heading and spikelets per spike). SNP 1A_539763488 was present in all high-yielding genotypes in intermediate and late-maturing groups, while 6B_682548960 was present in 11 intermediate-maturity genotypes out of 18. This high-yielding germplasm can be utilized in crossing programs targeting broad adaptation and grain yield improvement in the region. The information on the presence of the markers with significant effects on agronomic and adaptation traits can be utilized in the crossing and selection programs.

## 3. Discussion

### 3.1. Comparison of the Obtained GWAS Results with Previously Reported Genes and QTLs

Positional comparison of GWAS signals with previously reported genes and QTLs was performed using the IWGSC RefSeq v1.0 (Chinese Spring) reference genome.

5A_585020739_G_T is associated with plant height and days to heading. The allele increases both plant height (Effect = +3.10 cm, PVE = 31.9%) and the duration of the period to heading (Effect = +1.32…+1.80 days, PVE up to 59.4%). Its unidirectional effect points to the locus’s possible involvement in developmental regulator genes such as *Vrn-A1* or *Ppd-A1*. When breeding for early maturity, the accompanying reduction in height should be taken into account. SNP 5A_585020739_G_T (585.0 Mb) lies approximately 4.3 Mb from *Vrn-A1* (589.3 Mb), a key regulator of the vernalization response in wheat and an ortholog of Arabidopsis APETALA1 [13]. SNP 5A_586604587_G_A (586.6 Mb) lies approximately 2.7 Mb from *Vrn-A1*. Published estimates of genome-wide LD decay in bread wheat are often in the range of 2–5 Mb [14]. Therefore, treat *Vrn-A1* only as a nearby candidate gene.

SNP 1A_13767380_A_G (13.8 Mb) is located on the short arm of chromosome 1A. The classical *Rht* genes *Rht-B1* and *Rht-D1* are located on chromosomes 4B and 4D, respectively [15]. Thus, this signal is not explained by these classical *Rht* genes. Plant-height QTLs on 1A have been reported previously [16]. The large PVE (52.4%) and stability across four environments suggest a strong candidate locus, potentially representing a novel plant-height locus unrelated to the *Rht* family.

SNP 1A_539763488_C_T is associated with three spike-productivity traits. It affects grain weight per main spike (PVE up to 40.2%, Effect = +0.10…+0.12 g), spike weight (PVE up to 40.3%, Effect = +0.14…+0.19 g), and grain number per main spike (PVE = 21.7%, Effect = +1.84 grains). All effects are positive and stable across four environments.

The pleiotropic SNP 1A_539763488_C_T (539.8 Mb) is located on the long arm of chromosome 1A, in a region where QTLs for grain number per spike (498–499 Mb) [17] and grain weight (Qtgw.acs-1A.3) [18] have previously been described. The uniqueness of this locus lies in its simultaneous positive effect on three components of spike productivity, making it a priority target for marker-assisted selection. Pleiotropy of this kind is not unique to our panel: pleiotropic loci simultaneously affecting yield, height and phenology have likewise been reported in the ICARDA GWAS (e.g., on 1B, 2A, 3A and 5A) [19], and pleiotropic haplotype blocks associated with multiple yield and physiological traits were detected under heat–drought stress in Western Australian wheat [20].

SNP 5A_585412831_A_G had a divergent effect on the traits plant height and 1000-kernel weight. It exhibits antagonistic pleiotropy, reducing plant height (Effect = −6.19 cm, PVE = 44.0%, OMSK_2021) while simultaneously increasing 1000-kernel weight (Effect = +1.64 g, PVE = 27.1%, KRBK_2021). This is a valuable case for breeding, allowing selection of short-strawed forms with larger grain. 5A_585235173_C_T is associated with days to maturity and days to heading; PVE up to 55.4% for days to heading (ALLLOC_2022). Its MAF is 0.106—the allele is relatively rare, which may indicate the selective value of the alternative allele.

SNP 3A_732470863_C_T is associated with protein and gluten content and is located on chromosome 3A, one of the most significant QTL regions for protein content in wheat [21,22]—protein content (PVE up to 32.0%, Effect = +0.48…+0.62%) and gluten content (PVE up to 33.8%, Effect = +1.37…+1.39%); stable under Omsk conditions (OMSK_2020 and OMSK_ALLYEARS). The protein–gluten correlation is physiologically expected; however, identifying a shared genetic locus confirms the possibility of selecting for both traits simultaneously. Unlike the well-known major gene *Gpc-B1* (*NAM-B1*), located on chromosome 6BS [23]. However, this does not demonstrate an independent genetic factor; it may act through the same or a different pathway. We therefore describe it as a distinct genomic candidate rather than an established independent locus.

SNP 4B_9957885_T_G also affects protein and gluten content (PVE = 20.0% and 9.4%, respectively, OMSK_ALLYEARS). 7B_9702496_G_C is associated with days to heading and spikelets per main spike, with a positive effect on both traits. The relationship between phenology and spike architecture may reflect the influence of vegetative growth duration on the formation of spikelet number. 5B_601412050_A_G also affects days to heading and spikelets per main spike. This SNP showed a negative effect on both traits: the allele shortens the period to heading (Effect = −0.58 days) and reduces spikelet number (Effect = −0.31…−0.33). 1B_518180207_G_A is associated with 1000-kernel weight and grain width and has a positive effect on both traits: 1000-kernel weight (PVE = 24.0%, Effect = +2.53 g, OMSK_2020) and grain width (PVE = 41.1%, Effect = +0.067 mm, OMSK_2022). This allele is associated with the formation of larger and wider kernels. 6B_682550740_G_A affects grain perimeter and grain area, with a positive effect on both morphometric traits (OMSK_2020)—grain area: PVE = 37.9% and perimeter: PVE = 19.4%; this is a physically expected correlation. SNP 6B_682548960_T_C (682.5 Mb) is located on the long arm of chromosome 6B. Unlike the *TaGW2-6B* gene, located near the centromere [24], this locus is in the distal part of 6BL. The PVE (up to 48.6%) and stability across four environments indicate a large-effect candidate locus in this study. In the published study, a meta-analysis reported a stable grain-weight QTL on 6B (*Qtgw.acs-6B.1*) [18].

SNP 4D_509666790_G_A is associated with spike harvest index and grain width, with PVE up to 51.8% and PVE = 25.6%, respectively. It points to a possible link between kernel parameters and assimilate partitioning within the spike.

### 3.2. Practical Value for Breeding

This work identified the most promising loci for marker-assisted selection, including: 1A_539763488_C_T—simultaneous improvement of three components of spike productivity. It is recommended for pyramiding into breeding lines. 5A_585412831_A_G—a unique combination of “reduced height and increased 1000-kernel weight.” It is an ideal candidate for breeding short-strawed cultivars without loss of productivity. 5A_585020739_G_T and 1A_12366171_G_A—markers for controlling the days to maturity. Alleles with opposite effects make it possible to develop cultivars for both northern and southern regions. 3A_732470863_C_T and 4B_9957885_T_G—independent grain-quality loci (protein + gluten). Their pyramiding could produce an additive effect. 7B_9702496_G_C—owing to its exceptional stability and high allele frequency (MAF = 0.377), it could be incorporated into routine marker-selection schemes.

### 3.3. Limitations and Future Perspectives

Although the present study identified a set of stable and, in several cases, pleiotropic marker–trait associations, several limitations should be acknowledged when interpreting the results and planning their practical use.

First, the multilocation testing was confined to four main sites in Northern Kazakhstan and Western Siberia, and some environments had incomplete trait data in individual years. Consequently, the estimation of genotype-by-environment (G × E) interaction remains partial. The pronounced contrast among years (e.g., the heat and drought stress of 2020 versus near-average conditions in 2022) suggests that environment-specific QTLs may exist that were not detected in the multi-environment combined analysis. Future work could therefore extend phenotyping to a broader set of environments, including drought- and heat-stress trials, and apply G × E-aware models to dissect the stability and plasticity of the identified loci.

Second, although the KASIB panel is geographically and genetically diverse, it consists of 142 lines, which limits the statistical power to detect small-effect loci and rare alleles. Most of the significant SNPs explained only part of the phenotypic variance (PVE typically 10–50%), suggesting that a considerable proportion of the genetic control of these traits has yet to be resolved. Larger panels, as well as the integration of the present markers into genomic selection models, may help to account for the polygenic component of yield and quality. This limitation is shared across association studies: for example, in a 412-cultivar European GWAS of 24 DUS traits, no significant associations were detected for nine traits, largely owing to low heritability and complex genetic architecture [25], highlighting that both panel size and trait heritability jointly determine the power of GWAS.

Third, GBS provides a genome-wide but relatively low-density marker set, and the physical distances between the reported SNPs and the nearest candidate genes (e.g., ~4.3 Mb from *Vrn-A1*) are still considerable. Although the co-localization with previously described QTLs and known regulatory genes lends support to the biological relevance of the detected regions, the causal variants and genes remain to be identified. Fine-mapping in biparental populations derived from the superior lines characterized here, together with exome capture or whole-genome resequencing, could help narrow the confidence intervals and identify candidate genes.

Fourth, the loci reported for grain quality (3A_732470863_C_T and 4B_9957885_T_G) and plant height (1A_13767380_A_G) appear to be independent of the classical genes (*Gpc-B1*/*NAM-B1* and the *Rht* family, respectively). While this is of potential breeding interest, their effects would benefit from validation in isogenic or near-isogenic backgrounds before any large-scale deployment, as epistatic interactions with the major adaptation and dwarfing genes cannot be excluded.

Finally, the practical value of these findings is likely to depend on their adoption in regional breeding programs, where modern genotyping tools are not yet fully integrated. A practical next step is to develop and validate a cost-effective marker panel for the most promising SNPs (e.g., 1A_539763488_C_T, 5A_585412831_A_G, 5A_585020739_G_T, 3A_732470863_C_T, and 7B_9702496_G_C) and to test it on independent breeding material. Combining marker-assisted selection with genomic prediction, while pyramiding favorable alleles for spike productivity, reduced height, earliness, and grain quality, could provide a practical basis for accelerating the genetic improvement of spring wheat under the variable climatic conditions of the region.

## 4. Materials and Methods

### 4.1. Material

A KASIB core set of 142 genotypes (Table S4) was assembled from over 320 accessions exchanged among KASIB cooperators between 2001 and 2018 as described by Morgounov et al. (2024) [10]. The set comprised 59 entries from eight breeding programs in Kazakhstan and 83 entries from nine programs in Russia. Genotypes were chosen according to three criteria: (a) DNA diversity, characterized using 63 random KASP-SNP markers, with entries drawn from the distinct clusters defined by COREHUNTER software (v3); (b) geographic representation, ensuring that germplasm from every KASIB cooperator was included; and (c) agronomic performance, incorporating the five highest-yielding genotypes from each KASIB trial. The majority of the germplasm evaluated consisted of advanced lines, with cultivars making up approximately 35%. Kernel was first multiplied at Karabalyk Agricultural Experimental Station in 2018, then distributed to three additional sites and multiplied there in 2019, enabling replicated trials from 2020 onward, with each site using its own kernel.

### 4.2. GWAS Panel Yield Trials

Yield experiments were conducted at four sites: Omsk State Agrarian University named after P. Stolypin (55.025826° N and 73.310101° E; 3 m^2^ plots; 4 reps in 2020 and 2022, 2 reps in 2021), Karabalyk AES (53.844492° N and 62.130967° E; 3 m^2^ plots; 2 reps in 2020 and 2022, 3 reps in 2021), A.I. Barayev Research and Production Centre for Grain Farming (51.631622° N and 71.031765° E; 3 m^2^ plots; one rep in 2020 and 2022, 2 reps in 2021), and North Kazakhstan AES (54.165411° N and 69.553617° E; 5 m^2^ plots; two reps in 2021 and 2022). Trials followed a randomized complete block design with blocks laid out perpendicular to the main field gradient. Entries were randomized independently within each block. The following agronomic traits were recorded: days from emergence to heading and from heading to maturity, plant height (plot average), total and productive tillers per plant, spike length, grains per spike, 1000-kernel weight (1000KW), and grain weight per spike, following the methodology of Pask et al. [26] and using five random plants per replication. Trials were combined-harvested.

The experiments followed established local agronomy practices. The preceding crop was black fallow at all sites. Sowing was carried out at optimal dates between 15 and 25 May, with kernel rates ranging from 300 to 400 kernels per m^2^ depending on location. Weeds were controlled with common herbicides, and no disease or insect protection was applied. In keeping with standard post-fallow production practice, no fertilizers were used.

**Figure 3:**
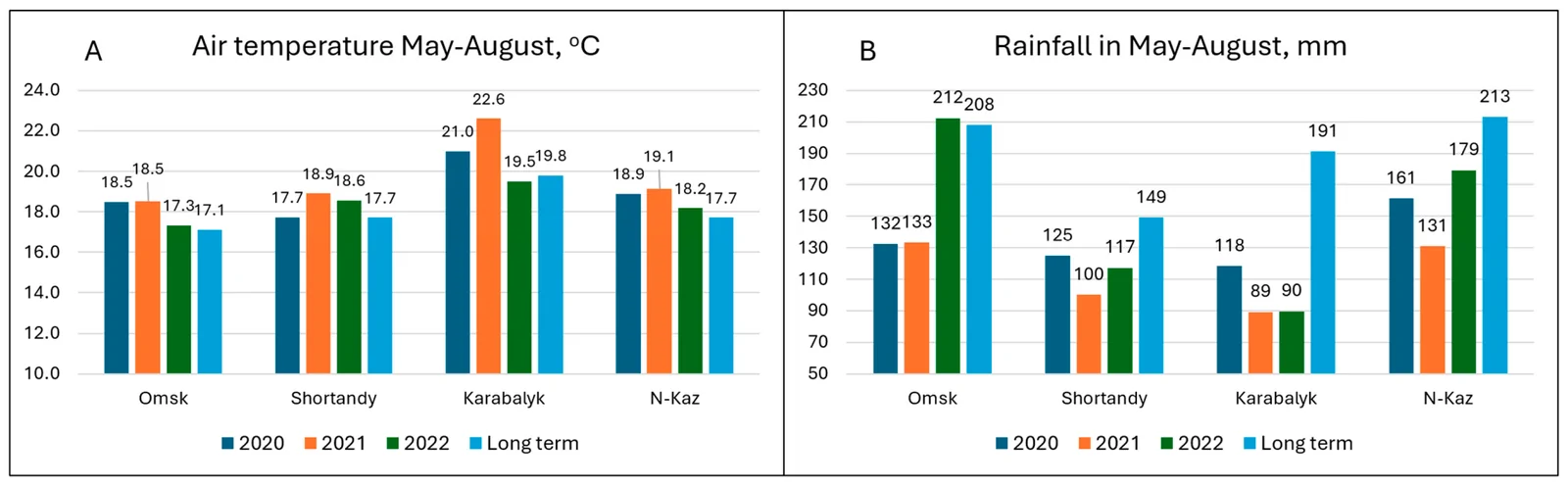
Air temperature (**A**) and precipitation (**B**) during the growing season at the experimental sites in 2020–2022.

The soils across the study sites were various chernozem types: ordinary (Karabalyk), leached (Shortandy and Shagalaly), and meadow (Omsk), with pH between 6.5 and 7.5, moderate phosphorus availability, and high potassium content; nitrogen supply was above average. Weather conditions varied over the three years (Figure 3). The season of 2020 was extremely dry and hot, with mean July temperatures 1–2 °C above long-term (2001–20) values. Karabalyk site also experienced moisture stress and heat in 2020 and 2022. Growing conditions in 2022 were close to the long-term average at Omsk, Shortandy, and Shagalaly. The only disease observed was stem rust at Omsk in 2020, which did not affect yield. Overall, the variable weather across the four sites and three seasons enabled a comprehensive evaluation of the germplasm.

### 4.3. Validation Yield Trials and Statistics

Validation of the SNPs’ effects on agronomic traits was conducted using regular KASIB trial data for genotypes that overlapped with the core set. These trials included 7 KASIB cycles: 2005–2006—13 entries overlapping with core set evaluated at 6–7 sites; 2007–2008—14 entries evaluated at 8 sites; 2009–2010—19 entries evaluated at 9 sites; 2011–12—22 entries at 9 sites; 2013–14—20 entries at 9 sites; 2015–16—23 entries at 9 sites; 2017–18—25 entries at 8 sites. Additional locations included in regular KASIB trials, as compared to GWAS panel experiments, included: Aktobe AES in Western Kazakhstan, Karagandy AES in Central Kazakhstan, Pavlodar AES in North-Western Kazakhstan, Chelyabinsk ARI and Kurgan ARI in the Southern Ural mountains, Omsk ARI and Altay ARI in Western Siberia. The soil and weather conditions varied greatly across sites and years. KASIB trials followed the same agronomy as described for GWAS panel trials with some local variations.

All statistical analyses were performed in the R environment (v4.3.3). Twenty-seven markers were subjected to quality control (removal of uninformative SNPs and markers with >20% missing data in the historical KASIB dataset), after which 23 informative SNPs remained and were carried forward for marker–trait association analysis. Marker–trait association analysis was used to determine whether a SNP marker was associated with agronomic traits. Practical significance was assessed quantitatively using PVE. Multiple comparison correlation and correlation analysis of phenotypic trends were used.

### 4.4. Genotyping, SNP Calling and GWAS Analysis

Genomic DNA was extracted from fresh young leaves (approx. 14 days after sowing) using the BioSprint^®^ 96 Plant Kit (QIAGEN, Hilden, Germany). The GBS (genotyping-by-sequencing) libraries were constructed as 96-plex following digestion with the restriction enzymes *PstI* and *MspI* at the Wheat Genetics Resource Center at Kansas State University (Manhattan, KS, USA). SNP calling was performed using TASSEL v.5.2.40 GBS v2 Pipeline [27] mapping on the Chinese Spring genome assembly (RefSeq v1.0) [28]. Quality filtering was then performed using PLINK 2.0 v2.00a6LM [29]. Variants with a genotype missing rate >5% were removed. Rare variants were excluded using a minor allele frequency (MAF) threshold of >8%. The observed heterozygosity of each marker was computed, and sites with an excess heterozygosity (>10%)—potentially reflecting paralogous or erroneously mapped loci—were removed. Missing genotypes were phased and imputed with Beagle 5.5 (build 27Feb25.75f) using only the study samples (no external reference panel).

To account for population structure, the imputed genotypes were pruned for linkage disequilibrium with PLINK 2.0 (window of 50 variants, step of 5, *r^2^* threshold of 0.1), retaining approximately independent markers. Principal component analysis was performed on these pruned markers, and the first ten principal components were used as covariates for the association analysis. Genome-wide association analysis was conducted in R with the GAPIT v3 package [30], using the BLINK (Bayesian-information-and-linkage-disequilibrium-iteratively-nested-key) mixed model [31]. Genotypes were encoded as 0/1/2 from the imputed VCF (Variant Call Format), and the ten principal components were supplied as fixed-effect covariates (CVs). The analysis was performed independently for each trait.

GWAS (genome-wide association study) was performed independently for each trait. For single-environment analyses, phenotypes were the observed values. For multi-environment datasets, adjusted means (best linear unbiased estimates, BLUEs) per genotype were estimated using a linear mixed model. Association statistics were computed for each SNP; the genomic control inflation factor (λ) was computed from the GAPIT *p*-values.

The significance threshold in GWAS was determined using a 1% false discovery rate (FDR) threshold to control for multiple testing. Effect sizes, standard errors, and the percentage of phenotypic variance explained (PVE) were extracted for all significantly associated SNPs.

Raw and filtered genotyping data, as well as GWAS results, are deposited on Zenodo [12].

## 5. Conclusions

This study identified a set of SNP markers with stable and often pleiotropic effects on the major agronomic, adaptive, and grain-quality traits of spring wheat in Northern Kazakhstan and Siberia. The most valuable loci include 1A_539763488_C_T, which simultaneously improved grain weight per spike, spike weight, and grains per spike; 5A_585412831_A_G, which combined reduced plant height with increased 1000-kernel weight; and independent protein- and gluten-enhancing loci on chromosomes 3A and 4B. Several phenology-associated markers mapped close to the vernalization gene *Vrn-A1*, whereas the height locus on 1A appears to lie in a region distinct from the classical *Rht* dwarfing genes, pointing to potentially novel genetic control. The independent validation of the main markers against more than a decade of multilocational KASIB trials confirms that their effects are robust across years, environments, and breeding backgrounds.

From a practical standpoint, these results provide regional breeders with a validated toolkit for marker-assisted and genomic selection. Because favorable alleles of the key loci are already present in elite, high-yielding lines within different maturity groups, the identified germplasm can serve directly as crossing parents. Pyramiding the pleiotropic productivity loci together with the quality and phenology markers should allow simultaneous improvement of yield potential, adaptation to the short and variable growing season, and grain protein and gluten content, while the phenology markers offer a means of tailoring season length to specific latitudes.

Future work should focus on fine-mapping and functional characterization of the causative genes underlying the most promising loci, particularly the novel plant-height region on chromosome 1A, and on field experiments that combine multiple favorable alleles to assess their additive and interactive effects. Broader integration of these validated markers into routine breeding, together with high-throughput phenotyping and deeper genotype-by-environment analyses, is expected to accelerate the release of better-adapted, higher-yielding cultivars and strengthen the stability of wheat production in this globally significant region.

## Figures and Tables

**Table 1:** SNPs with significant effect on agronomic traits in KASIB trials, 2020–22.

SNP	Trait	Site	Years	*p*-Value	MAF	Effect	PVE
1A_13767380_A_G	Plant height, cm	All	2020	1.227 × 10^−9^	0.127	−2.56	23.9
		All	2020–22	3.148 × 10^−10^	0.127	−2.91	32.7
		Omsk	2022	3.129 × 10^−8^	0.127	−4.33	28.3
		Omsk	2020–22	9.638 × 10^−14^	0.127	−4.60	52.4
6B_699553423_G_A	Plant height, cm	All	2020–22	4.316 × 10^−9^	0.151	−2.54	17.8
		Omsk	2022	1.181 × 10^−7^	0.151	−3.65	15.8
1B_15284504_C_T	Days to heading	Karabalyk	2021	2.695 × 10^−8^	0.313	−1.47	11.8
		Karabalyk	2020–22	7.181 × 10^−7^	0.313	−0.89	7.2
1B_603753334_T_G	Days to heading	Karabalyk	2020–22	2.370 × 10^−6^	0.123	−0.81	9.4
		N-Kaz	2022	9.417 × 10^−11^	0.123	−1.65	42.1
2B_691055986_T_G	Days to heading	Karabalyk	2021	2.980 × 10^−9^	0.085	2.24	32.4
		Karabalyk	2020–22	1.162 × 10^−6^	0.085	1.26	27.0
5A_585020739_G_T	Days to heading	All	2021	2.315 × 10^−10^	0.211	1.32	36.8
		All	2020–22	4.147 × 10^−16^	0.211	1.39	36.7
		Karabalyk	2021	4.665 × 10^−12^	0.211	1.61	26.4
		Omsk	2020	2.219 × 10^−21^	0.211	1.80	47.6
		Omsk	2020–22	2.247 × 10^−16^	0.211	1.60	59.4
	Plant height, cm	All	2021	2.586 × 10^−9^	0.211	3.10	31.9
7B_1026085_C_A	Days to heading	All	2021	1.741 × 10^−6^	0.127	−1.15	27.2
		All	2020–22	5.180 × 10^−7^	0.127	−0.85	18.3
		Karabalyk	2020–22	2.706 × 10^−7^	0.127	−0.88	13.3
5A_585235173_C_T	Days to heading	All	2022	2.632 × 10^−12^	0.106	1.64	55.4
	Days to maturity	Karabalyk	2020	6.367 × 10^−7^	0.106	1.44	12.3
5A_586604587_G_A	Days heading-maturity	All	2020–22	1.026 × 10^−12^	0.211	1.01	73.5
		Omsk	2020–22	4.713 × 10^−9^	0.211	1.22	69.9
1A_12366171_G_A	Days to maturity	All	2020	5.426 × 10^−9^	0.208	−1.13	51.2
		All	2020–22	6.154 × 10^−9^	0.208	−1.09	64.0
		Karabalyk	2020	1.308 × 10^−10^	0.208	−2.42	43.9
		Karabalyk	2022	1.554 × 10^−7^	0.208	−2.35	25.5
		Karabalyk	2020–22	2.696 × 10^−11^	0.208	−1.77	23.6
3D_73391771_A_G	Spikelets per spike	Omsk	2020	1.874 × 10^−6^	0.162	0.42	17.3
		Omsk	2022	1.655 × 10^−8^	0.162	0.58	37.5
4A_227089452_C_T	Spikelets per spike	All	2020–22	1.882 × 10^−8^	0.246	−0.36	22.8
		Omsk	2020–22	2.399 × 10^−6^	0.246	−0.31	26.5
5B_601412050_A_G	Spikelets per spike	All	2020–22	5.730 × 10^−7^	0.356	−0.33	10.5
		Omsk	2020	9.210 × 10^−7^	0.356	−0.32	4.9
		Omsk	2020–22	1.906 × 10^−6^	0.356	−0.31	9.5
	Days to heading	Omsk	2020	3.244 × 10^−6^	0.356	−0.58	4.7
7B_9702496_G_C	Spikelets per spike	All	2021	6.460 × 10^−7^	0.377	0.42	37.1
		All	2020–22	2.988 × 10^−10^	0.377	0.41	26.8
		Omsk	2020	2.210 × 10^−9^	0.377	0.37	8.1
		Omsk	2021	7.017 × 10^−9^	0.377	0.52	24.6
		Omsk	2022	4.140 × 10^−7^	0.377	0.41	14.8
		Omsk	2020–22	2.425 × 10^−9^	0.377	0.40	21.0
	Days to heading	All	2022	4.322 × 10^−8^	0.377	0.79	11.1
1A_539763488_C_T	Grain weight per spike, g	All	2021	1.632 × 10^−8^	0.144	0.10	40.2
		All	2020–22	4.227 × 10^−11^	0.144	0.10	35.5
		Omsk	2020–22	2.262 × 10^−8^	0.144	0.12	38.3
	Spike weight, g	All	2021	2.395 × 10^−8^	0.144	0.14	40.3
		All	2020–22	7.735 × 10^−9^	0.144	0.15	39.7
		Omsk	2021	1.763 × 10^−8^	0.144	0.17	18.3
		Omsk	2020–22	5.816 × 10^−13^	0.144	0.19	30.1
	Grains per spike	Omsk	2020–22	3.825 × 10^−6^	0.144	1.84	21.7
4D_509666790_G_A	Spike HI, %	All	2022	1.528 × 10^−7^	0.134	0.02	39.8
		All	2020–22	6.691 × 10^−10^	0.134	0.01	36.4
		Karabalyk	2021	3.350 × 10^−6^	0.136	0.03	22.3
		Karabalyk	2020–22	1.009 × 10^−10^	0.134	0.03	51.8
	Grain width, mm	Omsk	2021	1.520 × 10^−7^	0.134	0.05	25.6
1B_518180207_G_A	1000KW, g	Omsk	2020	4.995 × 10^−8^	0.095	2.53	24.0
	Grain width, mm	Omsk	2022	1.560 × 10^−7^	0.095	0.07	41.1
6B_119529954_G_A	1000KW, g	All	2020	3.621 × 10^−8^	0.134	−1.55	25.9
		Omsk	2020	7.003 × 10^−8^	0.134	−1.79	24.5
6B_682548960_T_C	1000KW, g	All	2020	4.915 × 10^−11^	0.384	−1.31	15.8
		All	2020–22	2.122 × 10^−9^	0.384	−1.13	24.8
		Omsk	2020	5.213 × 10^−9^	0.384	−1.41	18.0
		Omsk	2020–22	2.648 × 10^−8^	0.384	−1.33	48.6
5A_585412831_A_G	Plant height, cm	Omsk	2021	2.931 × 10^−12^	0.204	−6.19	44.0
	1000KW, g	Karabalyk	2021	6.773 × 10^−11^	0.204	1.64	27.1
6B_682550740_G_A	Grain perimeter, mm	Omsk	2020	5.088 × 10^−8^	0.391	0.20	19.4
	Grain area, mm^2^	Omsk	2020	1.092 × 10^−7^	0.391	0.36	37.9
3A_732470863_C_T	Protein content, %	Omsk	2020	4.081 × 10^−9^	0.183	0.62	21.3
		Omsk	2020–22	5.145 × 10^−9^	0.183	0.48	32.0
	Gluten content, %	Omsk	2020	1.628 × 10^−10^	0.183	1.39	33.8
		Omsk	2020–22	1.067 × 10^−10^	0.183	1.37	18.0
4B_9957885_T_G	Protein content, %	Omsk	2020–22	3.288 × 10^−6^	0.225	0.30	20.0
	Gluten content, %	Omsk	2020–22	1.225 × 10^−7^	0.225	0.89	9.4

Note: SNP—Single Nucleotide Polymorphism, MAF—Minor Allele Frequency, Effect—Effect of SNP, PVE—Proportion of Variance Explained.

**Table 2:** SNPs with validated effects on agronomic traits based on regular KASIB trials, 2005–2018.

SNP	Trait	Site	Years	MAF	Effect	PVE
1A_12366171_G_A	Days to heading	Karabalyk AES	2010	0.239	−6.1	59.2
	Grain yield, g/m^2^	Karabalyk AES	2017	0.239	−150.5	49.4
		Omsk SAU	2014	0.239	−63.3	58.6
			2016	0.239	−110.9	56.9
		Chelyabinsk ARI	2017	0.239	−150.6	60.8
1B_15284504_C_T	Days to heading	Karabalyk AES	2010	0.329	−5.1	53.8
	Grain yield, g/m^2^	Omsk SAU	2014	0.329	−632.7	58.6
5A_585412831_A_G	Days to heading	Kurgan ARI	2011	0.183	−10.25	71.4
		Aktobe AES	2011	0.183	−5.5	49.1
		Altay ARI	2011	0.183	−5.85	49.0
5A_586604587_G_A	Days to heading	Kurgan ARI	2011	0.188	−10.2	71.3
		Aktobe AES	2011	0.188	−5.5	48.7
		Altay ARI	2011	0.188	−5.6	52.6

Note: SNP—Single Nucleotide Polymorphism, MAF—Minor Allele Frequency, Effect—Effect of SNP, PVE—Proportion of Variance Explained.

**Table 3:** Grain yield, number of days to heading, 1000 KW and presence of SNPs with positive effects on agronomic traits in the top-yielding germplasm.

#	Genotype	Grain Yield, g/m^2^	Days to Heading	1000 KW, g	1A_12366171	1A_539763488	1B_15284504	5A_585412831	6B_682548960	7B_9702496
Early heading group (35–39.9 days)										
27	Novosib.18	273	39.6	34.3	-	-	-	-	+	-
78	Lut. 166-CП94	272	39.5	42.7	-	-	-	-	+	+
127	Lut. KS 963	270	39.8	33.6	-	het	het	-	-	-
38	Lut.1193	267	39.3	34.1	-	+	-	-	-	-
52	Lut.2102	263	39.7	38.1	-	+	-	-	-	+
Intermediate heading group (40–44 days)										
41	Saratov. 75	317	40.8	37.5	-	+	-	-	-	+
110	Lut.37-17	313	43.7	40.3	+	+	+	-	+	-
36	Ekada 113	311	42.5	39.5	-	+	-	-	+	-
126	Line-241-00-4	306	43.3	38.1	+	+	+	-	-	-
130	Erythrosp. 78	305	40.5	34.8	-	+	-	-	+	+
146	Lut.186/04-61	302	43.5	39.0	+	+	+	-	+	-
49	Lut.1764	300	43.0	38.4	-	+	-	+	-	+
132	Lut.126-05	299	41.2	36.7	-	+	-	-	+	-
70	Samgau	297	42.2	40.6	-	+	+	-	+	+
28	Lut. 307/97-23	297	43.6	36.8	-	+	-	-	+	+
148	SPChS 69	296	43.0	39.0	+	+	-	-	+	-
50	Lut.1082	296	43.4	40.0	-	+	-	+	-	+
116	OK-1	293	41.8	39.9	+	+	+	-	+	-
112	Lut.106-0/2003	293	42.2	36.3	-	+	-	+	-	-
68	Lutescens 9-33	290	42.0	37.2	-	+	-	-	+	+
115	A-125	290	41.0	35.2	-	+	-	-	+	-
107	Chebarkul.	288	42.1	38.7	-	+	+	-	+	-
1	Altay. zhnitsa	288	41.9	37.7	-	+	-	-	-	+
Late heading group (44–48 days)										
21	Zauljbinka	327	44.5	35.7	-	+	-	+	-	+
6	Tobol. 1	302	44.8	38.4	-	+	-	+	-	+
7	Lut.1012	301	44.5	37.9	-	+	-	-	+	+
69	Pavlodar. 11	298	44.3	34.6	-	+	-	+	-	+
40	LD-25	293	45.5	37.8	-	+	-	+	-	+
	LSD	40	1.7	2.1						

Note: SNP—1A_12366171, 1A_539763488, 1B_15284504, 5A_585412831, 6B_682548960, and 7B_9702496.

## Data Availability

The original contributions presented in this study are included in the article. Further inquiries can be directed to the corresponding authors.

## Supplementary Materials

The following supporting information can be downloaded at: https://www.mdpi.com/article/10.3390/ijms27219547/s1, there’re Table S1–S4 in Supplementary Materials. Full GWAS results (raw and QC-processed variants, Manhattan and QQ plots, tables containing *p*-values and effect sizes for each variant in GWAS) are deposited in Zenodo (https://zenodo.org/records/23162912) (accessed on 8 September 2026).

## Abbreviations

The following abbreviations are used in this manuscript:

SNP	Single Nucleotide Polymorphism
KASP	Kompetitive Allele-Specific PCR
GBS	Genotyping-by-Sequencing
QTL	Quantitative Trait Locus
GWAS	Genome-Wide Association Study
BLINK	Bayesian-information and Linkage-disequilibrium Iteratively Nested Keyway
PVE	Proportion of Variance Explained
TASSEL	Trait Analysis by aSSociation, Evolution and Linkage
PLINK	Whole Genome Association Analysis Toolset
GAPIT	Genome Association and Prediction Integrated Tool
